# The right to vaccination and the individual duty in collective health during a pandemic

**DOI:** 10.1016/j.clinsp.2022.100035

**Published:** 2022-05-31

**Authors:** Juliana Bertoldi Franco, Pilar Lecussan Gutierrez, Fábio Roberto Cabar, Chin An Lin

**Affiliations:** a Departamento de Odontologia, Instituto Central e Hospital Auxiliar de Suzano, Hospital das Clínicas, Faculdade de Medicina, Universidade de São Paulo, São Paulo, SP, Brazil; b Comitê de Bioética, Hospital das Clínicas, Faculdade de Medicina, Universidade de São Paulo, São Paulo, SP, Brazil; c Departamento de Obstetrícia e Ginecologia, Faculdade de Medicina, Universidade de São Paulo, São Paulo, SP, Brazil; d Medicina Interna Geral, Hospital das Clínicas, Faculdade de Medicina, Universidade de São Paulo, São Paulo, SP, Brazil

Beneficence, Nonmaleficence, Respect for Autonomy and Justice constitute the principles of Bioethics that permeate daily practice and defend the values of excellence in care and the relationship among patient, health team, family, and society. These principles must be defended by health institutions and professionals.^1^

Since it has started, the unprecedented public health crisis represented by the COVID-19 pandemic, the principles, and values of Bioethics has become increasingly important in the discussion of issues related to health care, such as resource allocation, end-of-life care, televisits, treatments not based on evidence, vaccine refusal, among others, are issues discussed so far.^2^

Vaccination is considered one of the greatest achievements in public health. Immunization programs have contributed to the decline in mortality and morbidity from infectious diseases and are responsible for the worldwide eradication of smallpox and polio. In order to achieve this success, high population adherence is necessary for the direct protection of vaccinated individuals, as well as high vaccination coverage rates, induce indirect protection, the so-called herd immunity.^3^

The anti-vaccine movements are seen in several countries around the world, especially regarding the application of vaccines in children, in which parents are responsible for this decision, with countless factors being used to justify the refusal of vaccination. In high-income countries with successful immunization programs and effective disease control, the fear of adverse reactions that the vaccine can cause is the major justification for the denial.^4^

The lack of parental trust in vaccines, for example, in the United States and the United Kingdom, is also linked to the many controversies and myths that have been brought to the population by the media and are currently maintained by anti-vaccine activists,^5^ such as the association of hepatitis B vaccines with multiple sclerosis, mumps, measles and rubella vaccine with autism, or even whooping cough vaccine leading to severe brain damage, seizures and mental disability. In adults, there is a supposed relationship between the tetanus vaccine and female sterilization. These associations, also called a conspiracy theory, caused a reduction in vaccination rates and later reflected in a significant increase in the number of cases and deaths worldwide.^3^

At the beginning of the COVID-19 pandemic, we had health professionals acting courageously inpatient care, developing health policy issues, and scientists working hard to develop vaccines to prevent COVID-19. Once vaccines proved to be safe and effective, their availability introduced a new ethical issue regarding the choice of people who would be immunized first. After the initial hysteria, the world faced cases of vaccine leftovers in developed countries due to population denials for vaccination, putting at risk the benefits that mass immunization provides and, consequently, pandemic control around the world.^6^

As harder this chaotic moment may be, vaccines developed for COVID-19 have the denial a part of the population against the existence of the disease, the forms of prevention, and the benefits of the vaccine. These justifications are related to government policy, unfounded beliefs about diseases and the benefits of vaccines, the power of social media, the spread of “fake news”, doubts about the insufficient time for doing research for safe vaccines omission bias, and coincidence bias.^3^^,^^7^

Jara et al.^8^ studied the effectiveness of the Coronavac vaccine in Chile in a population of about 10 million people. They found its effectiveness in preventing the disease in 65.9%, 87.5% in preventing hospitalization, 90.3% in preventing admission to the Intensive Care Unit, and preventable death in 86.3%. This study not only proves that vaccination is effective in protecting the individual but also shows that it can prevent serious forms of disease and death, which presents vaccination as the correct public health policy for the control of infectious and contagious diseases. Nonetheless, this result was only achieved because more than 79% of the adult population in Chile was vaccinated with two doses. Thus, the importance of massively vaccinating the population is highlighted.

Bioethics defends autonomy in a limited way, especially considering the autonomy of the individual versus the autonomy of the collective, causing harm to the vast majority. Nevertheless, it is important to guide the discussion that autonomy ends when an individual attitude brings potential harm to the community, mainly related to the sanitary control of diseases and devastating implications for humanity.^1^^,^^3^^,^^6^

The individual decision not to be vaccinated is a situation that fits in this context. Jara et al.^8^ showed that one of the most effective measures of pandemic control is mass vaccination. When people decide not to be vaccinated based on personal convictions, one of the pillars of the pandemic control measures is broken, which can bring irreparable health damage to them and everyone around them (family, friends, and colleagues from work, for example).

In Brazil, despite the Supreme Court (STF), in 2020, deciding that the vaccine is mandatory, not compulsory,^2^ it reported that restrictive (political, administrative, and sanitary) and educational measures could be adopted for people who insist on refusing to be vaccinated in order to protect the community.^9^

From a bioethical point of view, non-maleficence in many situations is more costly than beneficence. The latter denotes a proactive attitude toward doing good, while non-maleficence brings, in essence, a reflective attitude and limiting beneficence by reminding us that we cannot and must not cause harm, even if the intention is good.^1^

Hence, when the authors think about returning to daily activities, in a practical way, people not vaccinated by personal conviction (philosophical, religious, political, among others) should not be admitted to a hospital environment (as patient companion or visit, not to mention health care professionals), mainly in the wards. The population admitted to the hospital is fragile and vulnerable, making these individuals more susceptible to infection by agents such as SARS-CoV-2, causing damage to their clinical condition. The infection of these patients can be either direct, in the case of the person with the companion or visit, or indirectly, in cases of patients who are in the same hospital environment, such as in shared rooms.^4^^,^^5^

The denial of access to this group of people is a protective attitude both for them and for hospitalized patients since an unvaccinated person, once entering the hospital environment, can be infected by the virus circulating in that environment, as well as it can infect hospitalized patients. In this way, the principles of beneficence and non-maleficence are contemplated.

Broadening the scope of the discussion, still thinking about Health Institutions, Emanuel and Skorton^10^ discuss the vaccination of health workers and highlight three aspects:

1 Health professionals have an ethical duty and professional responsibility to protect others. The goals of healthcare workers are to promote the health and well-being of patients, families, inpatients, and the wider community. Getting vaccinated is one way to achieve this goal and protect the 'patients' health.
2 It is the duty and responsibility that health workers, so not only those on the front line, but also administrative workers and those who work to maintain the hospital's technical functionality and facilities, should be vaccinated to avoid compromising the health of everyone around.
3 Requiring healthcare employees to be vaccinated against COVID-19 is nothing new, it is an extension of well-established policies and practices around the world. Many healthcare facilities already required their employees to be vaccinated against hepatitis B, influenza, and other infectious diseases. As a result, health workers have historically been models of good behavior, especially in vaccination campaigns. In doing so, health workers provide good examples for the general population and show the importance of vaccination against COVID-19, and, ultimately, all vaccines.

Emanuel and Skorton^10^ even propose administrative measures against those who refuse to receive the vaccine (such as dismissal or suspension from office or removal from activities without remuneration), given the importance of the matter and its social impact on the control of pandemics.

## Conclusion

Although the anti-vaccine movement is growing, there is no evidence base since we know that big epidemics were and are still controlled with the use of vaccines. As much as fear and apprehension are relevant to the individual, common sense and clarity regarding the few adverse effects resulting from vaccination are negligible when compared with the harm caused by the disease in question.

Not authorizing the presence of unvaccinated people by conviction constitutes a bioethical practice, as it protects hospitalized patients, employees, and unvaccinated people. Vaccination is a right that must be assumed as a duty by everyone for the effective and collective control of the pandemic by COVID-19.

## Authors' contributions

Juliana Bertoldi Franco wrote the manuscript. Pilar Lecussan Gutierrez and Fábio Roberto Cabar reviewed the manuscript. Chin An Lin reviewed the manuscript and contributed to improving the manuscript.

## Declaration of Competing Interest

The authors declare no conflicts of interest.
